# Multiple primary tumours in women following breast cancer, 1973–2000

**DOI:** 10.1038/sj.bjc.6603172

**Published:** 2006-05-23

**Authors:** J S Raymond, C J R Hogue

**Affiliations:** 1 Rollins School of Public Health, Emory University, Atlanta, GA, USA

**Keywords:** breast cancer, multiple primary cancers, SEER

## Abstract

We investigated the predictors of the risk of developing a second primary cancer after breast cancer, this occurring in about 12% of affected women. The analysis included 335 191 females, registered in the National Cancer Institute's Surveillance Epidemiology and End Results (SEER) database, who had been diagnosed with breast cancer. Observed numbers of subsequent cancers in the SEER database with a first breast cancer diagnosed from 1973 to 2000 were compared with the expected numbers based on age-adjusted incidence rates to calculate standardised incidence ratios. Kaplan–Meier curves were conducted to determine the median time until the second primary cancer diagnosis. Average number of years until diagnosis varied by site and by age as well as median years until second cancer diagnosis. Most cancer risks decreased with age, but there was an increase in aging-related cancers such as lung cancer. The median years of follow-up were well beyond the 5-year mark. Breast cancer survivors should be advised of their increased risk for developing certain cancers in their lifetime.

Approximately, 600 000 women in the US are diagnosed with cancer in a given year, and 31% of them will be diagnosed with breast cancer. Breast cancer is the leading cancer for women, and deaths associated with breast cancer are second behind lung cancer and account for 15% of all cancer deaths (American Cancer Society, 2004). Survival rates (5, 10, and 20-year) continue to increase and many women will survive their breast cancer diagnosis and treatment (Rosen *et al*, 1989; Jones and Raghavan, 1993; American Cancer Society, 2004).

Second primary tumours may develop in women previously diagnosed with breast cancer. Yet, little is known about the risk of multiple primary tumours among breast cancer survivors. The purpose of this study was to examine whether women diagnosed with breast cancer have an increased risk of developing a subsequent cancer compared with women of equal age without an initial breast cancer diagnosis. The current study reports the most common second primary tumours following breast cancer, the time to diagnosis for women with a second tumour and how overall survival is affected for these women.

## MATERIALS AND METHODS

### Population

The study population was derived from the SEER Cancer Registry database, which encompasses approximately 11% of the national cancer burden (Fritz and Ries, 1998; SEER^*^Stat software, 2004). All patients in this study were women in the SEER Cancer Registry diagnosed with breast cancer between 1 January, 1973 and 31 December 2000. There were 335 191 women diagnosed with breast cancer (International Classification of Diseases for Oncology 2 (ICD-O-2) code C50) (Percy *et al*, 1990). Women were included if the breast cancer was categorised as *in situ* or malignant and if the morphology was either ductal, lobular, or medullary neoplasm (ICD-O-2 codes 8500–8540). A total of 2330 women developed a second cancer, but the information was not completely entered into the SEER database. These women were used in the survival analysis, because survival status information was provided, but these 2330 women were excluded from the incidence ratio analyses. Another 3177 women had missing follow-up time. These women were excluded from the survival analysis, but if they developed a second cancer simultaneously, they were then included in the incidence analyses. A total of 332 014 women were used in the survival analysis and were followed up until they were diagnosed with their second tumour, December 2000, or death before December 2000 whichever came first.

### Definition of second primary tumours

Multiple primary tumours are difficult to distinguish from metastatic tumours. The current study uses the SEER definition to define multiple primary tumours. The SEER definition takes into account, when determining multiple primary tumours, the site of the tumour, behaviour of the second tumour, histology, date of diagnosis, and the laterality if it is a paired organ (Fritz and Ries, 1998). Using this definition, metastatic and recurrent tumours are excluded from multiple primary tumour counts. There are exceptions and factors such as histology, laterality, and the date of diagnosis that can alter the decision of determining multiple primary tumours with paired organs. The SEER definition of multiple primary tumours for the breast comprises the following criteria:

- A single tumour with one histologic type is regarded as a single primary tumour; a single tumour disposed with multiple histologic types is regarded as a single tumour.
- A new tumour that contains the same histology as a previous cancer and is diagnosed within 2 months of the previous cancer is regarded as a single primary unless determined to be a recurrent or metastatic cancer.
- Simultaneous multiple tumours with the same histologic type within the breast are regarded as a single tumour, and even when different tumours have different behaviour codes.
- Simultaneous bilateral involvement of the breasts with only one histology is regarded as a single primary tumour.
- Multiple tumours with the same histologic type appearing in the breast and in a different site are regarded as multiple primary tumours unless stated as metastatic tumours.
- Multiple tumours of different histologic types within the breast are counted as multiple primary tumours regardless of time.
- Multiple tumours of different histologic types appearing in the breast and in a different site are multiple primary tumours regardless of time (Fritz and Ries, 1998; Howe *et al*, 2003).

### Analysis of risk of second primary tumours

The primary variable considered as the exposure in the first part of the analysis was the type of cancer the women developed after breast cancer. These women were then stratified into 10-year age groups for analysis. Age was determined by the age of each woman at the date of diagnosis for breast cancer. Data were analysed using STATA software (STATA Corporation, 2002). Risks are considered significant when corresponding 95% confidence interval did not include the null value. The risk was calculated for 14 second primary tumours in addition to an overall risk for second primary tumours in 10-year age periods. These were selected because there were at least 100 cases to analyse or showed to be statistically significant.

To control for potential confounding of the differences in age distributions, age-adjusted incidence rates were used for comparison. Age/period-specific cancer incidence rates from the SEER database were applied to the cohort, to calculate the number of subsequent tumours that would be expected for each site. This number was compared with the observed number to obtain a standardised incidence ratio (SIR) estimate and 95% confidence intervals (CI) were calculated assuming a Poisson distribution. Those sites that were statistically significant or showed an association with breast cancer were listed.

The cohort includes women diagnosed at earlier ages who survive cancer to the next 10-year age group. For example, women diagnosed before age 30 are included in older age groups if they did not have a second primary tumour diagnosis before age 30. Subsequent data accumulate cohorts similarly.

### Analysis of time to diagnosis for women with second primary tumour

For the women who were diagnosed with a second primary tumour, univariate analysis was performed using Kaplan–Meier curves, to estimate the time function diagnosis. Curves were calculated for all sites combined and for specific sites with at least 100 cases, and women were divided into decades of age at breast cancer diagnosis, 20–29, 30–39, 40–49, 50–59, 60–69, and 70 and older. Several exposure variables were explored in univariate analyses. Variables of interest were grouped and coded accordingly. These include multiple status, grade, AJCC stage or summary staging, and race. All variables were significant in the univariate analysis and included in the multivariate analysis. Multivariate analysis was determined between groups assessed by goodness of fit (GOF) methods (Kaplan and Meier, 1958; Lee, 1980; Snedecor and Cochran, 1980).

## RESULTS

Of those diagnosed with breast cancer during 1973–2000, 40 068 women developed one additional cancer after breast cancer, 3796 women developed two cancers, 351 women developed three cancers, 31 women developed four cancers, and 3 women developed 5 cancers. Of these women, 0.76% were diagnosed by age 29, 6.79% between 30 and 39, 19.72% between 40 and 49, 21.19% between 50 and 59, 22.34% between 60 and 69, and 29.20% at or after age 70. Second cancers were subsequently reported for 11.7% of the patients who were diagnosed with breast cancer before age 50, and 17% of patients diagnosed with breast cancer at or after age 50. Among women with a second tumour diagnosed, the mean time until the second cancer occurred was 6.2 years: 8 years for patients less than 50-years old and 5.7 years for women at or greater than age 50. Among women with a single breast cancer diagnosis, the average time from diagnosis of the breast cancer until death was 6.71 years for women less than 50-years old, and 4.5 years for women at or greater than age 50. 60.28% of the women were still alive at the end of follow-up.

The results of the standardised incidence ratio analysis are found in Table 1. For women diagnosed with breast cancer by age 29, the overall second primary tumour risk is large (SIR=17.3, 95% CI=13.7, 21.6), as is the risk of developing a second breast cancer (SIR=478.5) or stomach cancer (SIR=145.4). As age at primary diagnosis increases, the overall SIR's decrease until the eldest age group. Cancer sites with the highest ratios for women diagnosed with breast cancer before age 50 include bone, breast, leukaemia, lung, and ovary. However, having a breast cancer diagnosis was protective against cancer of the cervix for these age groups. For women who did not develop breast cancer until at least 50 years old, the overall SIR for developing a second cancer by age 59 was 2.7, which is much lower than risks among some of the earlier age groups. Again, the overall ratios decrease with age. Cancer sites with the highest SIR's were breast, colon/rectum, corpus uteri, lung, ovary, skin, and thyroid. Among older women, an initial breast cancer diagnosis was not protective against cervical cancer. Overall, both excess risk and protective effects decreased with age for most of the 14 cancers examined. For example, the SIR for a second breast cancer was greatest for women 20–29 years of age (478.53) and decreased dramatically and consistently after age 30 (Table 1).

Table 2 presents the data comparing the SIR's of a second breast cancer diagnosis with a second non-breast cancer diagnosis within 10-year time intervals. For women diagnosed with breast cancer by age 29, their risk of developing a second primary tumour other than breast within 10 years of their initial diagnosis is large (SIR=8.9, 95% CI=8.27, 9.60), but their risk for developing a second breast cancer, within the same 10 years, is five and a half times greater (SIR=48.4, 95% CI=46.5, 50.3). But after 20 years, these same women actually showed a protective effect against both cancer groups, breast and other, compared to the general population (SIR=0.2, 95% CI=0.2, 0.3; SIR=0.5, 95% CI=0.4, 0.6). For women diagnosed with breast cancer at or after age 70, their risk for developing either a second breast cancer or a second primary cancer, other than breast within 10 years, is virtually the same (SIR=1.84, 95% CI=1.8, 1.9; and SIR=1.6, 95% CI=1.5, 1.7). In general, most women had a more elevated risk of developing a second breast cancer, than a second primary cancer other than breast, but both risks were greater for these women than the general population. The risk reduced with time and age for all groups.

Table 3 summarises data from Kaplan–Meier Curves for time until second primary cancer diagnosis, stratified on age at diagnosis of the breast cancer and limited to the 12.3% of women who were diagnosed with a second primary tumour. The median years of follow-up until the second cancer is diagnosed ranged from 2.3 years for women over 70 developing cervix uteri to 18.08 for women 30–39 developing a second breast cancer. The difference in follow-up time for the age groups can be attributed to (1) a longer at-risk period for women <50 years for developing the second cancer and (2) the increased risk of developing some cancers at an advanced age.

**Figure 1:**
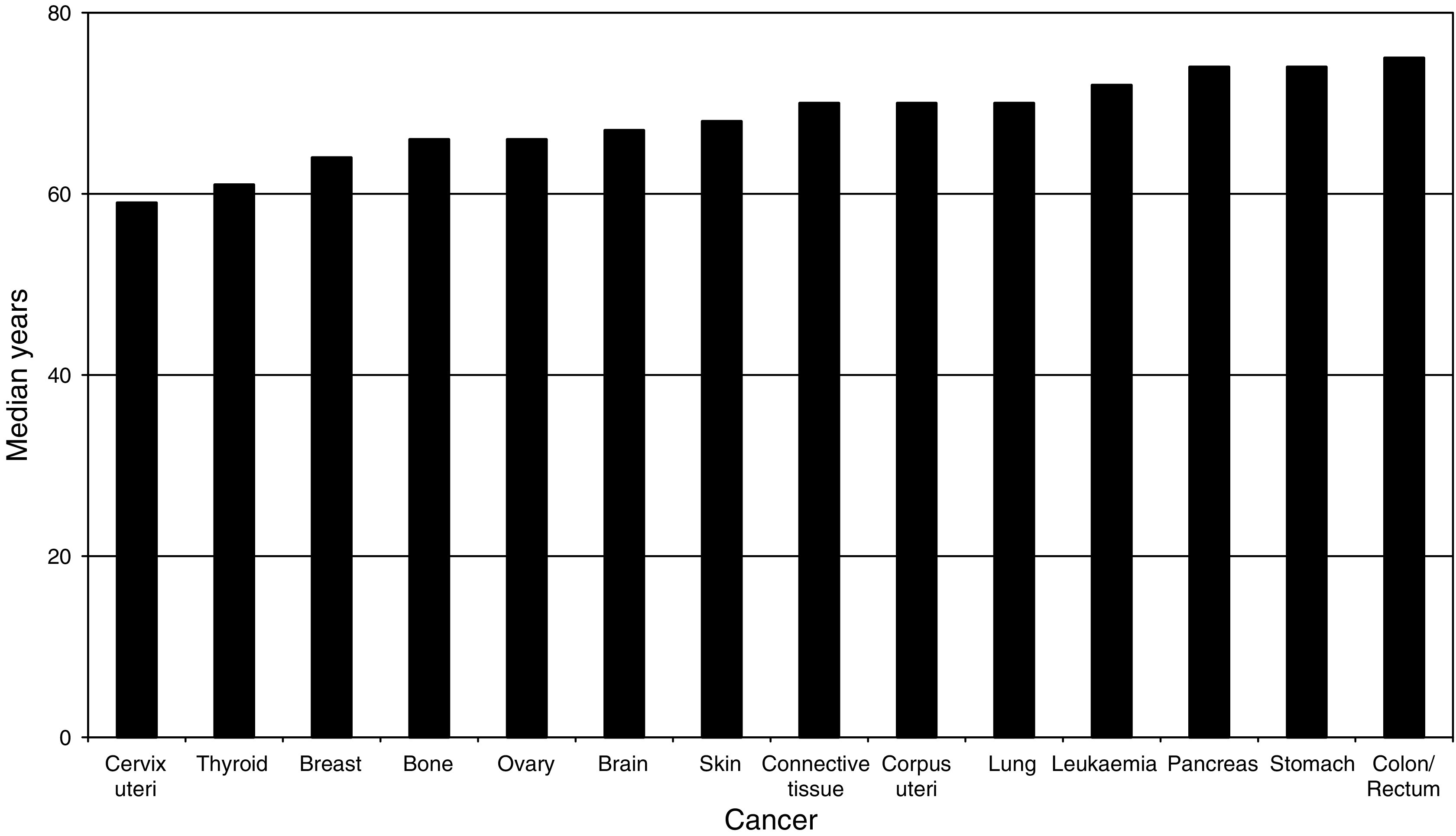
Median age at cancer diagnosis for second primary tumours.

Figure 1 illustrates the median ages at the time of the second cancer diagnosis for each of the 14 cancers. Cervix uteri cancer has the youngest median age with 59 years, while colon/rectum cancer has the oldest median age with 75 years.

**Figure 2:**
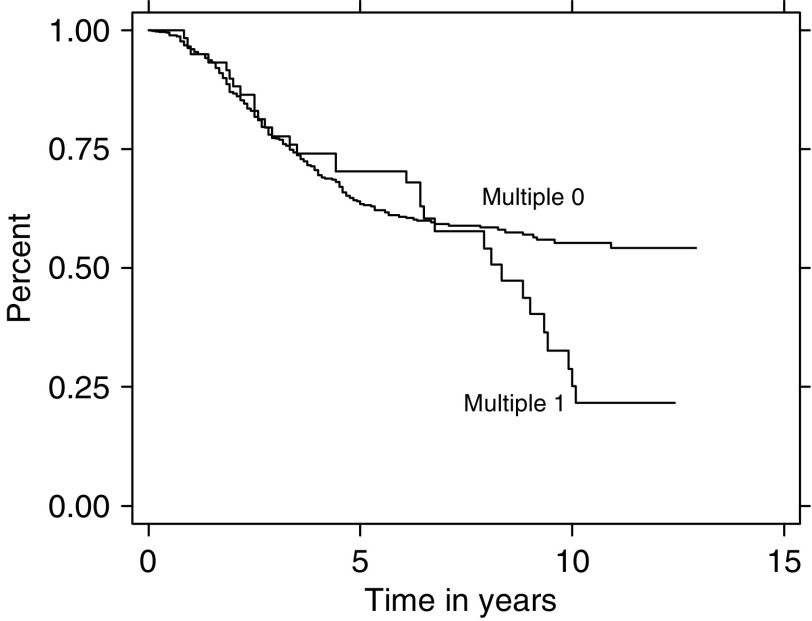
Survival analysis for women 20–29 years old diagnosed with breast cancer in or after 1988, stratified on multiple tumour status, controlling for race or grade.

Survival analysis was then constructed stratifying on the women who only developed breast cancer and those women who were diagnosed with breast cancer and a second primary cancer. Survival curves were adjusted for race and grade. In the 20–29 year old category, grade was categorised as an ordinal variable. Figure 2 illustrates survival analysis for women 20–29 years old diagnosed with breast cancer in or after 1988, stratified on multiple tumour status, controlling for race (*P*=0.621) and grade (*P*=0.511). The figure shows women with multiple primary cancers had poorer survival than women with only a breast cancer diagnosis over time. Women in this young age group who had metastatic breast cancer have a 15-year survival probability of only 23.23%.

In general, overall survival was poorer for women with multiple primary tumours compared with women with no second tumours. In addition to second tumours, grade of the breast cancer, race, and extent of disease played a role in determining the survival for each age group. A woman with a higher-grade breast tumour followed by a second tumour had much poorer survival than a woman with a low-grade breast tumour followed by a second tumour. This is also true for the extent of disease. Time also acted as a contributing factor to poorer survival.

## DISCUSSION

Changes in both the breast cancer incidence and the mortality rates have been documented in the past 25 years (Young *et al*, 2001). Evidence shows a fundamental increase in incidence, which is partly attributed to the improved screening and diagnostic techniques as well as to the changes in reproductive patterns, that is, women waiting until later to have children. Mortality rates had been relatively constant from 1973 to 1990. Since 1990, mortality rates have decreased by 2.3% overall. This decrease was more pronounced in women diagnosed with breast cancer before age 50 than with women aged 50 or older and have been attributed to the improvement of the various cancer treatments (Ries *et al*, 2003). Recent data have shown an overall survival rate for women diagnosed with breast cancer at least 5 years before to be as high as 87% but drops to 77% for the 10-year mark and to 52% by the 20-year mark (Ries *et al*, 2003). Given the increase in incidence and survival, it is important to assess health risks for breast cancer survivors.

Using a standard method, population-based data were collected from high-quality registries that covered approximately 11% of the US population. The study provided a unique opportunity to study a large number of women, over 335 000, across several years (1973–2000). This is the largest and most comprehensive study to date of cancer among breast cancer survivors. An international study (Chu *et al*, 1999) that examined the risk of a second primary tumour following an initial breast cancer diagnosis, only assessed risk for two age groups: <50 and ⩾50 years old; this also used the International Association for Research on Cancer definition of a second primary cancer, which is not widely used in the US. Another study examined women developing sarcoma after breast cancer, using the Surveillance, Epidemiology, and End Results (SEER) program data from 1973 to 1997 (Yap *et al*, 2002). The major drawback in this study was that it included only patients with invasive breast cancer.

This study found that 12.3% of breast cancer survivors were diagnosed with second primary malignant tumours among breast cancer survivors. Excess risks for 13 specific cancers were found, with the highest excess risks for breast. Among women with a second primary tumour, the time to second diagnosis varied by type of tumour and age at diagnosis of the first tumour. The results in this study are comparable to other findings regarding second primary breast cancer (Berstein *et al*, 2003). As expected, the risk of several cancers, including lung cancer, increased with age.

These results may help to inform patients and clinicians regarding expected quality of life for breast cancer survivors (Cimprich *et al*, 2002). A greater understanding of the patient's prognosis would assist both the patients and the clinician in treatment options. For example, if a woman understands her risks for developing a second primary cancer, she may choose to alter her treatment to be more aggressive in hopes of minimizing her risk. She may also need to be counselled regarding preventable risk factors, such as weight gain, that have been associated with premature mortality for women with a second breast cancer in other studies (Bernstein *et al*, 2002).

In this study, breast cancer survivors had the greatest risk for developing breast, bone, colon/rectal, connective tissue (sarcoma), leukaemia, lung, ovarian, and thyroid cancer. There are several possible explanations for these correlations, including genetic mutations. Four possible genetic mutations have been identified which can aid in predicting which women with breast cancer will develop multiple primary tumours. All four of these genetic mutations include breast cancer and other cancers. BRCA1 and BRCA2 are considered to be the breast cancer genes, but BRCA1 is also associated with ovarian cancer (Szabo and King, 1997). BRCA2 is associated with multiple primary tumours including breast, colorectal, ovarian and other cancers. Ovarian cancer, like breast cancer, is an oestrogen-driven cancer. Thus oestrogen is the most obvious link between the two cancers. However, the risk of ovarian cancer is also increased with the presence of the BRCA1 gene mutation (Szabo and King, 1997). Both BRCA1 and BRCA2 may bestow a heightened sensitivity to carcinogenic effects of radiation (Ford *et al*, 1994; Turner *et al*, 1999). Recently studies have shown a possible association between lung cancer and BRCA1 and BRCA2 mutation (Bergfeldt *et al*, 2000).

Multiple primary cancers, including acute adult leukaemia, have been associated with a family history of breast cancer (Rauscher *et al*, 2002). P53, known as Li–Fraumeni Syndrome, is associated with the development of cancer at a young age and is associated with breast cancer, sarcoma, brain cancer, and leukaemia (Lee, 1980). Women with P53 mutation have a lifetime probability of almost 100% for developing at least one of these cancers. Lastly the genetic mutation PTEN or Cowden Disease increases the risk for both breast and thyroid cancer.

Breast cancer survivors appeared to have protection from developing cervical cancer. Low socioeconomic status is a risk factor for cervical cancer while high socioeconomic status is a risk factor for breast cancer. However, the causal relationship underlying these associations is not well understood.

Breast cancer was associated with increased risk of connective tissue cancer for several of the age groups. Two possible explanations are (1) many women with breast cancer undergo radiation, which increases a woman's risk for connective tissue cancer in the surrounding areas and (2) some genetic mutations, including Li–Fraumeni Syndrome are linked with both breast cancer and connective tissue cancer (Malkin *et al*, 1990; Rubino *et al*, 2003).

Radiation may also increase the risk of lung cancer (Matesich and Shapiro, 2003; Roychoudhuri *et al*, 2004). During the 1950s, most of the upper body was exposed to radiation as the treatment for breast cancer. This exposure included the thyroid, lungs, breasts, and chest wall. This excess risk should therefore decrease as the cohort exposed to high doses of radiation doses ages. As well as radiation, chemotherapy as a treatment option for breast cancer may be a risk factor for developing leukaemia as a second primary cancer.

Among women who developed a second tumour, the time to cancer varied by age. Risk for cancer generally increases with age. This is most evident for the ‘older age’ cancers such as colorectal, in which median years to second diagnosis decreased from 18 for the 20–29 year group to less than four for the 70+ year group. Other age-dependent cancers were ovarian (median years ranging from 11 to less than 4) and pancreas (median years ranging from 15 to about 4). In the past, clinicians used as a general rule that if a woman is cancer free for 5 years after the initial diagnosis, then she is cured of cancer. This analysis shows that a sizeable proportion of women who developed a second tumour were cancer-free 5 years after their initial diagnosis. With the exception of the oldest group, the median time to diagnosis exceeded 5 years for the women who had a second tumour. For women who were diagnosed with breast cancer between the ages of 30 and 39, the median number of years until they were diagnosed with any second tumour was 11.42 years. This long lapse for the 30–39 year old age group is primarily due to the fact that the median number of years until a second breast cancer diagnosis was 18.08 years. It appears that the women in this age group are treated very aggressively and tend to have mastectomies performed on them. The only second cancer where the median number of years was less than 5 was cancer of the cervix uteri, but this estimate is probably unrealistic because of the small number of women who developed cancer of the cervix uteri after breast cancer. On the other hand, connective tissue cancer had a median number of years until diagnosis of over 10 years, except for the 70+ age group.

This study has several limitations. Although population-based cancer registries provide the opportunity to study these rare events, the registries lack some important variables such as smoking status, specific doses of treatment, and genetic make-up. Also, quality of data used for classifying multiple primary tumours is not under the control of the registries and may vary across site and over time. Even studies that used data sets known to be accurate found errors that affected the interpretation (Bergfeldt *et al*, 2000). Finally, missing and incomplete data may introduce measurement error. In this study, 5.7% of women known to have been diagnosed with a second tumour were excluded from the incidence risk analysis because of unknown diagnosis. If the distribution of the tumour sites in these women is substantially different from the distribution of known tumour sites, results for some of the more rare tumours may be biased. However, the number of unknown tumours is unlikely to have affected the risks from the more frequent tumours. Whatever bias, if present, is likely to be small, as there are striking similarities in results from published studies of selected second tumours (Yap *et al*, 2002).

In conclusion, it is clear that some women who survive breast cancer are at increased risk of additional cancers. Excess risks attenuate with increasing age at first diagnosis and with increasing years since first diagnosis. Excess risk also varies by cancer type, consistent with existing causal hypotheses regarding genetics mutations and risk of aggressive radiation therapy. More research should focus on isolating the factors that may predispose a breast cancer survivor to the development of subsequent cancers.

## Figures and Tables

**Table 1:** Standardized incidence ratios for a second primary cancer diagnosis by decade age at risk

	**20–29**		**30–39^a^**		**40–49**		**50–59**		**60–69**		**70+**		**50–59^b^**		**60–69^b^**		**70+^b^**			**Percent**
**Age/Cancer**	***N***	**SIR**	***N***	**SIR**	***N***	**SIR**	***N***	**SIR**	***N***	**SIR**	***N***	**SIR**	**N**	**SIR**	**N**	**SIR**	**N**	**SIR**	**Total**	**%**
All sites	78	**17.3**	1037	**5.9**	4547	**2.37**	2968	**1.64**					4720	**2.67**	10393	**1.74**	20506	**1.33**	44,249	
Breast	65	**478.5**	857	**21.8**	3463	**4.14**	1507	**2.19**	387	**1.45**			3118	**4.63**	5268	**2.83**	8262	**2.11**	22,927	51.81

*Other cancers*	13	**4.4**	180	**1.3**			1461	**1.3**	526	**0.9**	71	**0.8**	1602	**1.3**	5125	**1.3**	12244	**1.1**	21,322	48.19
Bone	1	**20.1**	3	**8.2**			7	**4.39**							11	**2.17**			22	0.05
Brain	1	**9.3**																	1	0.00
Cervix uteri			39	**0.5**	140	**0.55**	57	**0.68**	8	**0.35**							160	**0.72**	404	0.91
Colon/Rectum									82	**0.77**	11	**0.54**	214	**1.42**	869	**1.18**	3145	**1.07**	4,321	9.77
Connective tissue			6	**5.9**											49	**2.4**	105	**1.79**	160	0.36
Corpus uteri							189	**1.17**	49	**0.67**			207	**1.31**	679	**1.34**	1273	**1.61**	2,397	5.42
Leukaemia	3	**27.4**			69	**2.2**	72	**1.81**					64	**1.64**	259	**1.45**			467	1.06
Lung			17	**6.7**	127	**1.31**	228	**1.24**					303	**1.69**	978	**1.25**	1854	**1.12**	3,507	7.93
Ovary	2	**16**	27	**5**	153	**1.9**	141	**1.77**					147	**1.89**	338	**1.47**	531	**1.11**	1,339	3.03
Pancreas			3	**6.8**									112	**4.25**			488	**0.88**	603	1.36
Skin			15				104	**1.63**	32	**1.48**			107	**1.71**	264	**1.76**	559	**1.58**	1,049	2.37
Stomach	1	**145.4**	1												11	**0.14**			13	0.03
Thyroid			17	**1.9**			46	**1.64**					61	**2.23**	77	**1.53**	113	**1.38**	314	0.71

All numbers represents an elevated risk that is statistically significant by 95% confidence intervals.

a Women diagnosed before age 30 are included if they did not have a primary tumour diagnosis before age 30. Subsequent tables accumulate cohorts similarly.

b The first diagnosis of breast cancer occurred at or after age 50.

**Table 2:** Standardized incidence ratios categorised by either a second breast cancer diagnosis or a second primary cancer other than breast cancer, by time intervals after first diagnosis of breast cancer

	**Less than 10 years after initial breast cancer**				**10–19 years after initial breast cancer**				**20–29 years after initial breast cancer**			
	**Second primary breast cancer**		**Second primary non-breast cancer**		**Second primary breast cancer**		**Second primary non-breast cancer**		**Second primary - breast cancer**		**Second primary - non-breast cancer**	
**Age in years**	**SIR**	**95% CI**	**SIR**	**95% CI**	**SIR**	**95% CI**	**SIR**	**95% CI**	**SIR**	**95% CI**	**SIR**	**95% CI**
20–29	48.4	(46.5, 50.3)	8.90	(8.3, 9.6)	11.5	(10.6, 12.4)	1.46	(1.3, 1.2)	0.2	(0.2, 0.3)	0.5	(0.4, 0.6)
30–39	44.1	(42.3, 45.9)	2.79	(2.6, 3.0)	2.1	(1.9, 2.3)	1.35	(1.2, 1.5)	0.3	(0.2, 0.3)	0.3	(0.3, 0.4)
40–49	12.1	(11.6, 12.6)	4.08	(3.8, 4.3)	1.3	(1.2, 1.4)	0.88	(0.8, 1.0)	0.3	(0.2, 0.3)	0.0	(0.0, 0.05)
50–59	10.9	(10.6, 11.3)	4.75	(4.6, 5.0)	1.5	(1.4, 1.6)	0.99	(0.9, 1.1)	0.1	(0.1, 0.2)	0.2	(0.2, 0.2)
60–69	6.4	(6.2, 6.6)	2.99	(2.9, 3.1)	0.4	(0.3, 0.4)	0.50	(0.5, 0.5)	0.1	(0.1, 0.1)	0.1	(0.1, 0.2)
70+	1.8	(1.8, 1.9)	1.60	(1.5, 1.7)	0.2	(0.2, 0.2)	0.30	(0.3, 0.3)	0.1	(0.1, 0.1)	0.1	(0.1, 0.1)

**Table 3:** Point estimates for overall time until second primary cancer diagnosis, stratified on age, and limited to the 12.3% of women who were diagnosed with a second primary tumour

**Age at diagnosis**	**75%**	**50%**	**25%**
20–29	10.5	5.5	1.9
30–39	16.9	11.1	4.2
40–49	9.9	4.3	0.9
50–59	10.5	4.8	1.1
60–69	8.8	4.4	1.2
70+	6.0	2.7	0.4

## References

[bib1] American Cancer Society (2004). Available at http://www.cancer.org/, accessed 9 January 2004

[bib2] Bergfeldt K, Silversward C, Einhorn S, Hall P (2000) Overestimated risk of second primary malignancies in ovarian cancer patients. Eur J Cancer 36: 100–105 10741302 10.1016/s0959-8049(99)00244-0

[bib3] Bernstein JL, Lapinski R, Lynch C, Holford T, Thompson WD (2002) Factors influencing mortality among young women with second primary breast carcinoma. Cancer 95(10): 2051–2058 12412157 10.1002/cncr.10950

[bib4] Berstein JL, Lapinski RH, Takore SS, Doucette JT, Thompson WD (2003) The descriptive epidemiology of second primary breast cancer. Epidemiology 14(5): 552–558 14501270 10.1097/01.ede.0000072105.39021.6d

[bib5] Chu KU, Turner RR, Hansen NM, Brennan MB, Bilchik A, Giuliano AE (1999) Do all patients with sentinel node metastasis from breast carcinoma need complete axillary node dissection? Annals of Surgery 229(4): 536–541 10203087 10.1097/00000658-199904000-00013 PMC1191740

[bib6] Cimprich B, Ronis DL, Martinez-Ramos G (2002) Age at diagnosis and quality of life in breast cancer survivors. Cancer Prac 10(2): 85–93 10.1046/j.1523-5394.2002.102006.x 11903273

[bib7] Ford D, Easton DF, Bishop DT, Narod SA, Goldgar DE (1994) Risks of cancer in BRCA1 mutation carriers. Lancet 343: 692–695 7907678 10.1016/s0140-6736(94)91578-4

[bib8] Fritz A, Ries L (eds) (1998) SEER Program Code Manual, 3rd edn. Bethesda: National Cancer Institute

[bib9] Howe HL, Weinstein R, Hotes J, Kohler B, Roffers SD, Goodman MT (2003) Multiple primary cancers of the ovary in the United States, 1992–1997. Cancer 10(Suppl): 2660–2675 10.1002/cncr.11348 12733131

[bib10] Jones VE, Raghavan D (1993) Quantum leaps in treatment of high-risk breast cancer? Prove it!. Eur J Cancer 10: 1488–1493 10.1016/0959-8049(93)90027-d 8398281

[bib11] Kaplan EL, Meier P (1958) Non-parametric estimation from incomplete observations. J Am Stat Assoc 53: 457–481

[bib12] Lee ET (1980) Statistical Methods for Survival Analysis. Belmont, CA: Lifetime Learning Publications

[bib13] Malkin D, Li FP, Strong LC, Fraumeni Jr JF, Nelson CE, Kim DH, Kassel J, Gryka MA, Bischoff FZ, Tainsky MA (1990) Germ line p53 mutations in a familial syndrome of breast cancer sarcomas, and other neoplasms. Science 250: 1233–1238 1978757 10.1126/science.1978757

[bib14] Matesich SM, Shapiro CL (2003) Second cancers after breast cancer treatment. Semin Oncol 30(6): 740–748 14663775 10.1053/j.seminoncol.2003.08.022

[bib15] Percy C, Van Holten V, Muir C (eds) (1990) International Classification of Disease for Oncology 2nd edn. Geneva: World Health Organization

[bib16] Rauscher GH, Sandler DP, Poole C, Pankow J, Mitchell B, Bloomfield CD, Olshan AF (2002) Family history of cancer and incidence of acute leukemia in adults. Am J Epidemiol 156(6): 517–526 12225999 10.1093/aje/kwf075

[bib17] Ries LAB, Eisner MP, Kosary CL, Hankey BF, Miller BA, Clegg L (2003) SEER Cancer Statistics Review, 1975–2000. Bethesda, MD: National Cancer Institute

[bib18] Rosen PR, Goshen S, Saigo PE, Kinne DW, Hellman S (1989) A long-term follow-up study of survival in stage I (T1N0M0) breast carcinoma. J Clin Oncol 7: 355–366 2918331 10.1200/JCO.1989.7.3.355

[bib19] Roychoudhuri R, Evans H, Robinson D, Moller H (2004) Radiation-induced malignancies following radiotherapy for breast cancer. Br J Cancer 91(5): 868–872 15292931 10.1038/sj.bjc.6602084 PMC2409877

[bib20] Rubino C, de Vathaire F, Shamsaldin A, Labbe M, Le MG (2003) Radiation dose, chemotherapy, hormonal treatment and risk of second cancer after breast cancer treatment. Br J Cancer 89(5): 840–846 12942115 10.1038/sj.bjc.6601138 PMC2394476

[bib21] Snedecor GW, Cochran WG (1980) Statistical Methods 7th edn. The Iowa State University Press: Ames, IA

[bib22] Surveillance Research Program (2004) National Cancer Institute SEER*Stat software (version 5.0). Available at http://www.seer.cancer.gov/seerstat

[bib23] STATA Corporation (2002) STATA Statistics/Data Analysis, Version 7.0

[bib24] Szabo CI, King MC (1997) Population genetics of BRCA1 and BRCA2. Am J Hum Genet 60: 1013–1020 9150148 PMC1712447

[bib25] Turner BC, Harrold E, Matloff E, Smith T, Gumbs AA, Beinfield M, Ward B, Skolnick M, Glazer PM, Thomas A, Haffy BG (1999) BRCA1/BRCA2 germline mutations in locally recurrent breast cancer patients after lumpectomy and radiation therapy: implications for breast-conserving management in patients with BRCA1/BRCA2 mutations. J Clin Oncol 17: 3017–3024 10506595 10.1200/JCO.1999.17.10.3017

[bib26] Yap J, Chuba PJ, Thomas R, Aref A, Lucas D, Severson RK, Hamre M (2002) Sarcoma as a second malignancy after treatment for breast cancer. Int J Radiat Oncol Biol Phys 52(5): 1231–1237 11955733 10.1016/s0360-3016(01)02799-7

[bib27] Young Jr JL, Roffers SD, Ries LAG, Fritz AG, Hurlbut AA (eds) (2001) SEER Summary Staging Manual – 2000: Codes and Coding Instructions. Bethesda, MD: National Cancer Institute, NIH Pub. No. 01-4969

